# Ethanol sclerotherapy of head and neck venous malformations

**DOI:** 10.1590/S1679-45082014AO2844

**Published:** 2014

**Authors:** José Luiz Orlando, José Guilherme Mendes Pereira Caldas, Heloisa Galvão do Amaral Campos, Kenji Nishinari, Mariana Krutman, Nelson Wolosker

**Affiliations:** 1 Hospital AC Camargo, São Paulo, SP, Brazil; 2 Hospital das Clínicas, Faculdade de Medicina, Universidade de São Paulo, São Paulo, SP, Brazil; 3 Hospital Israelita Albert Einstein, São Paulo, SP, Brazil

**Keywords:** Venous malformations/therapy, Sclerotherapy, Ethanol/therapeutic use, Sclerosing solutions/therapeutic use, Head/blood supply, Neck/blood supply

## Abstract

**Objective:**

This retrospective study evaluated the results of sclerotherapy with low doses of ethanol for treatment of head and neck venous malformations.

**Methods:**

We treated 51 patients, 37 females. Median age was 23 years. Patients were treated with percutaneous intralesional injection of alcohol every two weeks and followed up prospectively for a median period of 18 months. Most lesions affected the face and cosmetic disfigurement was the most frequent complaint.

**Results:**

We performed a median of 7 sessions of sclerotherapy. Complete resolution or improvement was observed in 48 patients presented. Five cases of small skin ulceration, two cases of hyperpigmentation and two of paresthesia were documented; all of them were treated conservatively.

**Conclusion:**

Percutaneous sclerotherapy with low doses of ethanol is a safe and effective treatment modality for venous malformations affecting the head and neck.

## RESUMO

**Objetivo:**

Estudo retrospectivo que analisou os resultados da escleroterapia, com doses baixas de etanol, realizada sob anestesia local para tratamento de malformações venosas na cabeça e pescoço.

**Métodos:**

Foram tratados 51 pacientes, sendo 37 do gênero feminino. A mediana de idade foi de 23 anos. Os pacientes foram tratados com injeções alcoólicas nas lesões, por via percutânea, em sessões quinzenais, e acompanhados prospectivamente por um período mediano de 18 meses. A maioria das lesões acometia a face e a queixa principal mais frequente foi deformidade estética.

**Resultados:**

Foram realizadas, em média, sete sessões de escleroterapia. Resolução completa ou melhora foi observada em 48 pacientes. Cinco pacientes apresentaram pequena úlcera cutânea, dois hiperpigmentação e dois parestesia, sendo todos tratados conservadoramente.

**Conclusão:**

A escleroterapia percutânea com doses baixas de etanol é um método seguro e eficaz para tratar malformações venosas na cabeça e pescoço.

## INTRODUCTION

Venous malformations (VM) account for two thirds of all vascular malformations and consist of spongy clusters of veins of varying size, venules and venular capillaries.^(1)^ VM may be located anywhere in the body and present as well-defined or diffuse lesions.^(2)^

VM may be present at birth and grow slowly as the individual develops. The diagnosis is based on clinical history and physical examination findings.^(3)^ Unlike hemangiomas, that tend to grow rapidly and then stabilize or involute spontaneously, spontaneous involution of VMs has not been reported.^(4)^

Upon physical examination,^(5,6)^ VMs appear as purple or bluish compressible tumor-like formations devoid of arterial murmur or beat. Clinical manifestations of head and neck VMs^(7,8)^ include cosmetic disfiguring, pain, ulceration, bleeding, compression of nerves or adjacent structures and functional impairments.

Magnetic resonance imaging is a useful complementary diagnostic and follow-up tool in VM cases.^(9,10)^ On T2-weighted and inversion recovery sequences, VM appear as hyperintense channels or septated areas that may contain low fluid levels and pheboliths (signal voids).

Ultrasonography is a simple non-invasive method employed to differentiate high flow from low flow lesions or to guide percutaneous injections during sclerotherapy procedures.^(11,12)^

Percutaneous intralesional injection of liquid sclerosing agents is the therapeutic method of choice for VMs and is usually performed under general anesthesia.^(5,13)^ Pure alcohol is the most powerful and effective sclerosing agent available;^(14,15)^ however, local and systemic complications may occur when high doses are used.^(16–18)^

Good clinical results have been reported following outpatient treatment of limb VM with multiple injections of low doses of ethanol.^(17,18)^

## OBJECTIVE

To evaluate the results of percutaneous sclerotherapy with low doses of ethanol in patients with head and neck venous malformations.

## METHODS

This study was approved by the Ethics Committee of *Hospital das Clínicas de São Paulo* (online protocol number 8,536). Fifty-one patients, 37 (72.6%) female, aged between 6 and 80 years (median age: 23 years) were studied. They were treated with percutaneous ethanol sclerotherapy under local anesthesia on outpatient basis between July 1995 and June 2007 and retrospectively analyzed. Patient follow-up ranged from 6 to 72 months (median follow-up period: 18 months). Due to the retrospective nature of the study, informed consent was not required.

Clinical data of the 51 patients studied are given on table 1.

**Table 1:** Clinical characteristics

Clinical data		n (%)
Site of lesion		
	Face	36 (70.6)
	Tongue	6 (11.8)
	Neck	5 (9.8)
	Lip	4 (7.8)
Symptoms		
	Deformity	39 (76.4)
	Pain	10 (19.6)
	Bleeding	2 (4)
Previous treatment		
	None	42 (82.4)
	Surgery or embolization	9 (17.6)

Most lesions affected the face and cosmetic disfigurement was the most frequent complaint. Most patients had not received previous treatment.

Lesions were measured (mm) along their longer axis using an ordinary ruler and assigned to one of three groups according to size: small (up to 3cm; 21 patients), medium-sized (between 3 and 15cm; 21 patients) or large (greater than 15cm; 9 patients).

Patients were submitted to alcohol percutaneous injection every two weeks. Injection site was selected by palpation and corresponded to the area where venous dilation was largest. The injection technique consisted of percutaneous puncture of the lesion^(19)^ with a 21-gauge butterfly needle introduced perpendicularly to the skin surface and into the anomalous venous space. Blood reflux confirmed proper needle positioning. Iodinated contrast media was then slowly and gradually injected into the lesion under fluoroscopic guidance, and opacification of the anomalous space monitored. Following description of VM characteristics and quantification of the volume drained into the venous system, percutaneous injection of approximately 2mL of 2% lidocaine hydrochloride followed by pure ethanol (1 to 3mL for small and medium-sized lesions and up to 5mL for large lesions) was performed. Additional small volumes of local anesthetic were injected just before needle withdrawal and a slightly compressive dressing applied. Patients remained under observation for approximately 30 minutes before discharge.

Data on clinical progression of symptoms and lesion size following sclerotherapy, the number of sessions required per group and sclerotherapy related complications were collected. Patient satisfaction was graded using a straightforward questionnaire: patients were asked to grade their respective clinical improvement on a 1-to-4 scale, in which 1 equals little or no improvement, 2 moderate improvement, 3 significant improvement, and 4 complete remission of symptoms. In this study, scores 2 and 3 were grouped and classified as improvement.

## RESULTS

Treatment results were evaluated based on patient's satisfaction and reduction in lesion size. Complete remission of symptoms was reported by 17 (33.3%) patients and improvement by 31 (60.8%), with no changes in the remaining 3 (5.9%) patients. Lesions regressed completely in 14 (27.4%) patients, decreased in 34 (66.7%) and remained unchanged in 3 (5.9%) patients.

Data on lesion size progression are displayed on table 2.

**Table 2:** Lesion size progression

Group	Size reduction (partial or complete) n (%)	Unchanged n (%)
Small	21 (100)	0 (0)
Medium	20 (95.2)	1 (4.8)
Large	7 (77.8)	2 (22.2)

Partial or complete reduction of VM lesions in most patients across all groups suggested percutaneous ethanol sclerotherapy is effective regardless of lesion size.

The number of percutaneous ethanol sclerotherapy sessions per group is given in table 3.

**Table 3:** Number of sessions

Group	n	Faixa de variação	Mediana
Small	21	2–19	4
Medium	21	4–47	5
Large	9	6–36	17

**Figure 1:**
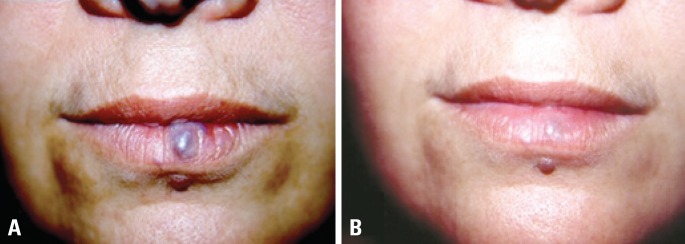
Small facial lesion before (A) and after (B) three sessions of ethanol sclerotherapy

**Figure 2:**
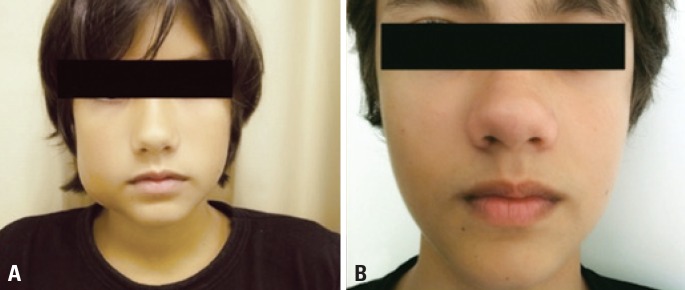
Medium-sized facial lesion before (A) and after (B) four sessions of ethanol sclerotherapy

**Figure 3:**
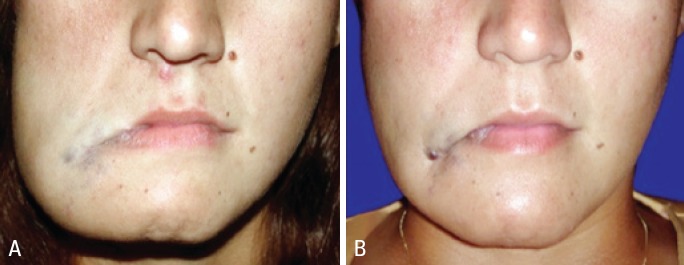
Large facial lesion before (A) and after (B) 27 sessions of ethanol sclerotherapy

The number of sclerotherapy sessions performed in this study ranged from 2 to 47 (median of 7 sessions) and was directly related to lesion size. Of the three patients who did not respond to treatment, one had medium-sized and two had large lesions. The number of sessions in these patients ranged from 13 to 36 (median of 22). Clinical progression of small, medium-sized and large lesions, and the respective number of treatment sessions are illustrated in figures 1, 2 and 3.

There were no complications in 42 patients (82.4%), while 5 (9.8%) presented a small skin ulcer, two (3.9%) presented hyperpigmentation and two (3.9%) presented transitory paresthesia. All complications were treated conservatively with good evolution.

## DISCUSSION

VM are the most common symptomatic low flow vascular malformations affecting the human body, particularly the head and neck.^(6,13)^

VM can be treated in several ways, such as irradiation, cryotherapy, laser therapy, surgical excision and sclerotherapy.^(2)^ Laser therapy is effective for small superficial VMs^(20)^ and surgical resection for localized well-defined lesions.^(21)^ Extensive lesions are difficult to demarcate during surgery and radical excision is associated with significant functional impairments, cosmetic disfigurement and high recurrence rates.^(22)^

Sclerotherapy is an effective treatment modality for VM and can be performed with a variety of sclerosing agents. Pingyangmycin^(23)^ (bleomycin hydrochloride), a chemotherapy drug used to treat oral cancer, is currently being employed as a sclerosing agent.

Sodium tetradecyl sulphate (sotradecol) and polidocanol have detergent properties and interfere with cell surface lipids causing endothelial damage,^(24)^ with resulting thrombosis and fibrosis. However, marked recanalization of treated lesions has been reported^(14,25)^ and anaphylactic shock and blindness may occur following sotranecol injection of extensive cephalic VM.^(6,13)^

Pure ethanol is a potent, cheap and widely available fibrosing agent with well-known and controllable side effects.^(26)^ Direct contact of ethanol with the vascular endothelium promotes denaturing of blood proteins, vessel wall necrosis and disruption of erythrocytes, with subsequent thrombosis and fibrosis of the intima leading to regression of the VM.^(27)^

The sclerosing dose of ethanol is strongly correlated with serum levels of ethanol in treated patients. Ethanol sclerotherapy toxic effects are directly related to the volume injected into the organism and doses above 1,0mL/kg may cause respiratory depression, cardiac arrhythmia, rhabdomyolysis and hypoglycemia.^(14,17)^ In this study, maximum doses of 5mL of pure ethanol proved insufficient to produce toxic side effects.

Most sclerotherapy studies to date involve large doses of ethanol injected under general anesthesia and report several complications, such as immediate VM thrombosis and venous circulation blocking resulting in severe edema,^(5)^ superficial thrombophlebitis, deep vein thrombosis with or without associated pulmonary embolism,^(18)^ cardiac arrest^(14)^ and trophic cutaneous scars or lesions.^(28)^

In a study by Berenger et al.^(5)^ involving 40 patients treated with large doses of ethanol, 30 patients (75%) showed marked improvement or complete cure while 10 (25%) had slight improvement or did not respond to treatment. Major complications reported included acute blistering (50%), hemoglobinuria (28%), deep ulceration (13%), and nerve injury (7.5%). Transient facial paresis was reported in two patients and permanent unilateral vocal cord paralysis in one.

Lee et al.^(15)^ followed 87 patients after sclerotherapy with large doses of ethanol (305 sessions in total; mean 3.5). Outcomes were excellent in 23 (32.4%) patients, good in 37 (52.1%) and poor in 11 (15.5%). Patients presenting swelling and pain at the injury site were treated with intravenous or intramuscular analgesic drugs. Other complications reported (four patients; 4.6%) were respiratory distress (two cases), tongue hypoesthesia (one case) and transient facial nerve paralysis (one case).

In a study by Liu et al.^(23)^, 23 patients were treated with low doses of ethanol and followed-up for 20 months in average. All patients had remission or relief of symptoms. Excellent or good clinical results were obtained in 9 and 14 patients, respectively. Patients showing mild to moderate swelling and pain were treated conservatively and recovered within a few days. Skin necrosis or nerve damage was not reported.

In this study, 96.1% of the patients had complete remission or improvement of symptoms. Partial reduction in lesion size or complete resolution was obtained in 94.1% of patients, with minor complications that responded well to conservative treatment. Percutaneous sclerotherapy performed on outpatient basis was therefore considered an effective treatment for VMs.

## CONCLUSION

Percutaneous sclerotherapy with low doses of ethanol under local anesthesia is a safe and effective treatment for head and neck venous malformations.

## References

[1] Paes E, Vollmar J (1995). Diagnosis and surgical aspects of congenital venous angiodysplasia in the extremities. Phlebology.

[2] Dompmartin A, Vikkula M, Boon LM (2010). Venous malformation: update on aetiopathogenesis, diagnosis and management. Phlebology.

[3] Finn MC, Glowacki J, Mulliken JB (1983). Congenital vascular lesions: clinical application of a new classification. J Pediatr Surg.

[4] Serra AM, Soares FM, Cunha AG, Costa IM (2010). Abordagem terapêutica dos hemangiomas cutâneos na infância. An Bras Dermatol.

[5] Berenguer B, Burrows PE, Zurakowski D, Mulliken JB (1999). Sclerotherapy of craniofacial venous malformations: complications and results. Plast Reconstr Surg.

[6] de Loriemier AA (1995). Sclerotherapy for venous malformations. J Pediatr Surg.

[7] Kim KH, Sung MW, Roh JL, Han MH (2004). Sclerotherapy for congenital lesions in the head and neck. Otolaryngol Head Neck Surg.

[8] Lee CH, Chen SG (2005). Direct percutaneous ethanol instillation for treatment of venous malformation in the face and neck. Br J Plast Surg.

[9] Dubois J, Soulez G, Oliva VL, Berthiaume MJ, Lapierre C, Therasse E (2001). Soft-tissue venous malformations in adult patients: imaging and therapeutic issues. Radiographics.

[10] Memis A, Arkun R, Ustun EE, Kandiloglu G (1996). Magnetic resonance imaging of intramuscular haemangiomas with emphasis on contrast enhancement patterns. Clin Radiol.

[11] Dubois J, Patriquin HB, Garel L, Powell J, Filiatrault D, David M (1998). Soft-tissue hemangiomas in infants and children: diagnosis using Doppler sonography. AJR Am J Roentgenol.

[12] Donnelly LF, Bissett GS, Adams DM (1999). Combined sonographic and fluoroscopic guidance: a modified technique for percutaneous sclerosis of low-flow vascular malformations. AJR Am J Roentgenol.

[13] Siniluoto TM, Svendsen PA, Wikholm GM, Fogdestam I, Edström S (1997). Percutaneous sclerotherapy of venous malformations of the head and neck using sodium tetradecylsulphate (sotradecol). Scand J Plast Reconstr Surg Hand Surg.

[14] Burrows PE, Mason KP (2004). Percutaneous treatment of low flow vascular malformations. J VascInterv Radiol.

[15] Lee IH, Kim KH, Jeon P, Byun HS, Kim HJ, Kim ST (2009). Ethanol sclerotherapy for the management of craniofacial venous malformations: the interim results. Korean J Radiol.

[16] Yakes WF, Luethke JM, Parker SH, Stavros AT, Rak KM, Hopper KD (1990). Ethanol embolization of vascular malformations. Radiographics.

[17] Orlando JL, Caldas JG, Campos HG, Nishinari K, Wolosker N (2010). Outpatient percutaneous treatment of deep venous malformations using pure ethanol at low doses under local anesthesia. Clinics (Sao Paulo).

[18] Orlando JL, Caldas JG, Campos HG, Nishinari K, Wolosker N (2010). Ethanol sclerotherapy of superficial venous malformation: a new procedure. Dermatology.

[19] Boxt LM, Levin DC, Felows KE (1983). Direct puncture angiography in congenital venous malformations. AJR Am J Roentgenol.

[20] Derby LD, Low DW (1997). Laser treatment of facial venous vascular malformations. Ann Plast Surg.

[21] Ethunandan M, Mellor TK (2006). Haemangiomas and vascular malformations of the maxillofacial region-a review. Br J Oral Maxillofac Surg.

[22] Kane WJ, Morris S, Jackson IT, Woods JE (1995). Significant hemangiomas and vascular malformations of the head and neck: clinical management and treatment outcomes. Ann Plast Surg.

[23] Liu Y, Liu D, Wang Y, Zhang W, Zhao F (2009). Clinical study of sclerotherapy of maxillofacial venous malformation using absolute ethanol and pingyangmycin. J Oral Maxillofac Surg.

[24] Pascarella L, Bergan JJ, Yamada C, Mekenas L (2005). Venous angiomata: treatment with sclerosant foam. Ann Vasc Surg.

[25] Mimura H, Kanazawa S, Yasui K, Fujiwara H, Hyodo T, Mukai T (2003). Percutaneous sclerotherapy for venous malformations using polidocanol under fluoroscopy. Acta Med Okayama.

[26] Ellman BA, Green CE, Eigenbrodt E, Garriott JC, Curry TS (1980). Renal infarction with absolute ethanol. Invest Radiol.

[27] Berthelsen B, Fogdestam I, Svendsen P (1986). Venous malformations in the face and neck. Radiologic diagnosis and treatment with absolute ethanol. Acta Radiol Diagn (Stockh).

[28] Lee KB, Kim DI, Oh SK, Do YS, Kim KH, Kim YW (2008). Incidence of soft tissue injury and neuropathy after embolo/sclerotherapy for congenital vascular malformation. J Vasc Surg.

